# Genetic Variation and Genotype by Environment Interaction for Agronomic Traits in Maize (*Zea mays* L.) Hybrids

**DOI:** 10.3390/plants11111522

**Published:** 2022-06-06

**Authors:** Mohammad Ashraful Alam, Marufur Rahman, Salahuddin Ahmed, Nasrin Jahan, Mohammad Al-Amin Khan, Mohammad Rafiqul Islam, Amnah Mohammed Alsuhaibani, Ahmed Gaber, Akbar Hossain

**Affiliations:** 1 Spices Research Centre, Bangladesh Agricultural Research Institute, Bogura 5810, Bangladesh; a.alam_83@yahoo.com; 2 Regional Station, Bangladesh Institute of Research and Training on Applied Nutrition, Rangapur 5470, Bangladesh; marufur@birtan.gov.bd; 3 Maize Breeding Division, Bangladesh Wheat and Maize Research Institute, Dinajpur 5200, Bangladesh; su_ahmed66@yahoo.com; 4 Plant Genetic Resources Research Centre, Bangladesh Agricultural Research Institute, Gazipur 1701, Bangladesh; nasrin.jahan83@gmail.com; 5 Regional Spices Research Centre, Bangladesh Agricultural Research Institute, Gazipur 1701, Bangladesh; mithunkhan93@gmail.com; 6 Regional Agricultural Research Station, Bangladesh Agricultural Research Institute, Pabna 6620, Bangladesh; rafiq_bari2@yahoo.com; 7 Department of Physical Sport Science, College of Education, Princess Nourah bint Abdulrahman University, P.O. Box 84428, Riyadh 11671, Saudi Arabia; amalsuhaibani@pnu.edu.sa; 8 Department of Biology, College of Science, Taif University, P.O. Box 11099, Taif 21944, Saudi Arabia; a.gaber@tu.edu.sa; 9 Department of Agronomy, Bangladesh Wheat and Maize Research Institute, Dinajpur 5200, Bangladesh

**Keywords:** genetic diversity, trait association, GCV, genetic variability, genetic advance, heritability, PCV

## Abstract

In order to develop high-yielding genotypes of adapted maize, multilocation trials of maize were performed including forty-five maize hybrids exploiting genetic variability, trait associations, and diversity. The experiments were laid out in an RCB design and data were recorded on eight yield and yield-contributing traits, viz., days to anthesis (AD), days to silking (SD), anthesis–silking interval (ASI), plant height (PH), ear height (EH), kernels per ear (KPE), thousand-kernel weight (TKW), and grain yield (GY). An analysis of variance (ANOVA) showed significant variation present among the different traits under study. The phenotypic coefficient of variance (PCV) showed a higher value than the genotypic coefficient of variance (GCV), indicating the environmental influence on the expression of the traits. High heritability coupled with high genetic advance was found for these traits, indicative of additive gene action. The trait associations showed that genotypic correlation was higher than phenotypic correlation. Based on genetic diversity, the total genotypes were divided into four clusters, and the maximum number of 16 genotypes was found in cluster IV. Among the eight yield and yield-contributing traits, PH, ASI, EH, and TKW were the important traits for variability creation and were mostly responsible for yield. Genotypes G5, G8, G27, G29, and G42 were in the top ranks based on grain yield over locations, while a few others showed region-centric performances; all these genotypes can be recommended upon validation for commercial release. The present findings show the existence of proper genetic variability and divergence among traits, and the identified traits can be used in a maize improvement program.

## 1. Introduction

Maize (*Zea mays* L.) is a popular staple cereal after rice and wheat [1,2]. It is also used as a raw material for human food, as well as animal feed products. The nutrition quality of maize (i.e., starch, protein, oil, fiber, sugar, and ash) is very rich [3]. In addition, corn oil and corn flakes are popular across the globe [3]. China, Brazil, Argentina, and Ukraine are the best maize-producing countries, and the USA is ranked first in maize production [4]. 

In Bangladesh, maize stands in the second position for production after rice and is ranked third as a staple cereal [5]. The last decade’s maize production increased three times in Bangladesh, and surprisingly, the growth rate per year is 11.40% [5]. Although the maize production area has expanded and the adaptation rate is high, we are still behind in achieving sustainable food security with the existing available commercial cultivar. Hence, it is urgent to create high-yielding new varieties of maize to break the yield ceiling. It is most important to enhance the qualities of yield-contributing traits to develop the best variety. Yield is a polygenic and complex trait and is related to other yield-contributing traits that are easily inherited [2,3]. The genetic diversity, variability, and heritability inside the current germplasm are key thrusts that improve the efficiency of the breeding program [1,6,7]. 

Hence, trait selection for any crops solely based on the heritability of the particular traits sometimes may lead to an incorrect choice, where considering genetic advances along with it might be more effective. Heritability explains the degree of extent for heritable traits from parents to segregants; in addition, genetic advance is a powerful tool in searching for the original advance predicted under selection [8]. Correlation and path analysis provide relationships among the traits and also show the importance of a trait in contributing to the yield. Traits having high values of the genotypic coefficients of variations indicate that the traits are highly heritable and have good potential for perfect selection [9,10,11]. 

Along with trait selection, breeders have also intensely emphasized the development of stable genotypes for different climatic conditions and locational variations. Those varieties are suitable for a wide range of planting. The ideal variety should have a high mean yield with low fluctuations in diverse environments and locations [12,13], although region-centric varieties can meet the demand of a specific region. The responses of genotype and location interactions on yield and yield-attributed traits have been well-recognized for a while. Improvement is possible either by reducing the genotype × location interaction through breeding for a region-centric adaptation or by identifying a germplasm having wide adaptation from selection across varying environments. Apart from adaptivity, a genotype × location interaction study can also provide information about similarities of locations for a variety of responses that may help in making decisions for adaption targets and test sites [14].

Considering the above aspects, a few attempts have already been made to enhance the trait qualities of maize in Bangladesh. The present investigation is undertaken to screen variability, genetic advance, trait associations, and diversity among the genotypes and traits of maize for the development of high-yielding cultivars suitable for different locations.

## 2. Materials and Methods

### 2.1. Plant Genetic Materials

Forty-five test crosses of maize hybrids were used in the present study. A list and the pedigrees of the test crosses of hybrids are given in Table S1. The female parents were developed locally at the Ishwardi location, comprising a screened sample of the population pool, and they were further bulked for one more season. The male counterparts were collected from the plant-breeding division of the Bangladesh Agricultural Research Institute (BARI), followed by three more seasons of regeneration. A large number of the test crosses were made at the Ishwardi location, but only a few hybrids were included in the study (only those successful hybrids representing a female parental line with all three male inbred lines).

### 2.2. Experimental Site and Design

The present investigation was carried out at three regional stations (Barishal, Ishwardi, and Jashore) of the Bangladesh Agricultural Research Institute (BARI) during 2015–2016. Among these locations, the Barishal region is under the agro-ecological zone of the Ganges Tidal Floodplain. The soil type is noncalcareous grey floodplain with 15–20 inches of total annual rainfall and a tropical monsoon climate with 25.6 °C as the average temperature. The Ishwardi location is under the active Brahmaputra-Jamuna Floodplain zone. The soil type is sandy and silty alluvium. The yearly maximum temperature is 36.8 °C, and the minimum temperature is 9.6 °C with an annual rainfall of 1872 mm. The regional station at Jashore is under the AEZ High Ganges River Floodplain. The soil in the AEZ falls under dark grey calcareous floodplain soil. The annual average temperature ranges from 15.4 to 34.6 °C with rainfall of 60.5 inches. Details of the weather that prevailed during the cropping season at the studied locations are given in Table S2. In all three locations, all the materials were arranged in a randomized complete block design (RCBD) and repeated three times.

### 2.3. Experimental Details

The unit plot size was 7.5 m^2^. The distance from row to row was 75 cm, and the plant-to-plant distance was 25 cm. Standard intercultural operations were performed during plant production. Well-decomposed farmyard manure (FYM) at 6 t ha^−1^ was applied one week before sowing, and a mixture of N:P:K at 120:60:40 kg ha^−1^ was mixed into the soil immediately before sowing. During the growth stages of the crop, two hand weedings were conducted, one at 18 days after sowing (DAS), while the second one was performed at 36 DAS. Earthing-up was conducted two times during the whole cropping cycle. A total of three irrigations (i.e., one at the vegetative stage, the second during anthesis (to avoid pollen desiccation), and the third during the grain-filling stage) were applied during the whole cropping cycle. For plant protection, one spray was applied against leaf feeders during the late vegetative stage.

### 2.4. Evaluation of Agronomic Traits

Data on different traits were collected according to the standard methods stated in the IBPGR [15]. Data on the days to anthesis (AD), days to silking (SD), anthesis–silking interval (ASI), plant height (PH), ear height (EH), kernels per ear (KPE), thousand-kernel weight (TKW), and grain yield (GY) were recorded for all three locations. Data on yield and yield-related traits were collected during the flowering-to-harvesting stage. In each replication, 3 plants were selected to collect these yield and yield-related traits. 

### 2.5. Statistical Analysis

An analysis of variance (ANOVA) for all the recorded data and mean separation tests at the 5% and 1% levels of probability were performed using SAS software (version 9.2). The details of the analysis were as follows.

#### 2.5.1. Analysis of Variance

The analysis of variance for individual traits was carried out using R software version 4.1.2 [16]. The linear model of observations in an alpha lattice design was as follows:

Yij=μ+ti+rj+eij 

where *Yij* is the observed trait’s value for *i*th treatment at the *j*th replicate; *ti* is the fixed effect of the *i*th treatment; *rj* is the effect of the *j*th replicate; and *eij* is the experimental error.

ANOVA for yield was performed for genotype × location using the R platform [16] and the ‘Plant breeding’ package software [17]. 

The model was written as below:

Yij=μ+gi+lj+gi×lj+εij 

where *Yij* is the observed mean yield for *i*th genotype at the *j*th location; *gi* is the genotype effect; *lj* is the effect of the location; *gi* × *lj* is the interaction effect of *i*th genotype at the *j*th location; and *εij* is the residual error.

Pi is the phenotypic index, which was estimated as:

Pi = the mean of a particular genotype over all the locations − the grand mean of all genotypes over all locations, and

Li is the locational index, which was estimated as: 

Li = the mean of all the genotypes in a particular location − the grand mean of all genotypes over all locations.

#### 2.5.2. Variability Estimates

Variability estimates including genotypic and phenotypic variances, heritability, genotypic and phenotypic coefficients of variations, and genetic advance were estimated according to [18,19,20,21].

##### Phenotypic and Genotypic Variance

These parameters were calculated according to the formula given by [21] for genotypic variance:

$$\delta^{2}g=\frac{\mathrm{MSG}-\mathrm{MSE}}{r}\times 100$$

where MSG is the mean sum of square for the genotypes; MSE is the mean sum of square for the error; and *r* is the number of replications.

The phenotypic variance was calculated as follows:

$$\delta^{2}p=\delta^{2}g+\delta^{2}e$$

where $\;\delta^{2}g$ is the genotypic variance, and $\delta^{2}e$ is the environmental variance equal to the mean square error.

The genotypic and phenotypic coefficients of variation were calculated with the following formula [22]:

$$\mathrm{GCV}=\frac{\delta_{g}\times 100}{\bar{x}}$$

$$\mathrm{PCV}=\frac{\delta_{p}\times 100}{\bar{x}}$$

where GCV is the genotypic coefficient of variation; PCV is the phenotypic coefficient of variation; $\;\delta_{g}$ is the genotypic standard deviation; $\;\delta_{p}$ is the phenotypic standard deviation; and $\bar{x}$ is the population.

##### Estimation of Heritability

Heritability, in a broad sense for all the traits, was computed as suggested by [21]:

$$h^{2}\;\%=\frac{\sigma^{2}{}_{g}}{\sigma^{2}{}_{p}}\times 100$$

where h^2^ is heritability in a broad sense; *σ*^2^*_g_* is the genotypic variance; and *σ*^2^*_p_* is the phenotypic variance.

The heritability was classified as low (0–30%), moderate (30–60%), or high (>60%), as suggested by [23].

##### Estimation of Genetic Advance

The genetic advance was calculated as follows:

GA = K·h^2^·*σ_p_*

or as genetic advance:

$$\mathrm{GA}=K\;\frac{\sigma^{2}{}_{g}}{\sigma^{2}{}_{p}}\cdot \sigma_{ph}$$

where K is the selection intensity, or the value that is 2.06 at a 5% selection intensity; *σ_ph_* is the phenotypic standard deviation; h^2^ is heritability in a broad sense; *σ*^2^*_g_* is the genotypic variance; and *σ*^2^*_p_* is the phenotypic variance.

##### Association Analysis

To observe associations among the studied traits, a correlation analysis was performed with R software [16] using the ‘Agricolae’ package [24].

##### Regression Analysis

A multiple regression analysis was performed to determine the extent of the relationship between GY with the other studied traits. Stepwise regression was also performed to find the most-contributing traits to GY in different locations. The analysis and visualization were performed using the ‘ggplot2’ [25] package in R software [16].

##### Grouping or Clustering

Grouping for all the genotypes was conducted using a cluster analysis, as suggested by D2 analysis [26,27]. The grouping method divided the genotypes into more or less homogeneous groups. The grouping of traits was also helpful to find the closeness of the traits. The analysis and visualization were performed with R software [16].

## 3. Results and Discussion

The study was conducted to discover the variations of yield and yield-contributing traits in forty-five maize genotypes. Data for eight traits were collected from three locations (Barishal, Ishwardi, and Jashore) and statistical analyses were conducted for probable explanations. The ANOVA of eight yield and yield-related traits of maize is shown in Table 1. The data in Table 1 showed significant (*p* ≤ 0.05) variations among the genotypes for AD, SD, ASI, PH, EH, KPE, TKW, and GY at the three locations.

### 3.1. Genetic Variability among Genotypes

The variations among the tested genotypes for the target traits allowed for the selection of desirable genotypes for future crop improvement. In the current study, the differences among the genotypes in response to eight traits under three locations were explained, and the results are shown in Table 1. It was noted that the minimum number of days for AD and SD were required at the Barishal (88.03 and 89.12 days, respectively) location, followed by Jashore (92.33 and 93.55 days, respectively). The ASI and PH showed almost similar values at the three locations. In the cases of EH (119.18), KPE (510.99), and TKW (352.83), the maximum values were found at the Barishal location, and the lowest values for KPE (465.02) and TKW (326.60) were observed at the Ishwardi location. Considering the grain yield, the maximum value was recorded at Ishwardi (11.65), and the minimum was found at Jashore (10.51). The expression of every trait depends on the interaction between genes and environmental factors. Sometimes, more environmental influences hinder the expression of traits. The variances due to genotype and phenotype indicate the contribution of the heritable part within a trait-based phenotypic expression. In the present study, the phenotypic variance appeared to be higher than the genotypic variance for all the traits under the three different locations for all the genotypes (Table 1). This information suggested that the environmental impact on the phenotypic expression of genes is controlled by these traits. Previous results of some researchers [28,29,30] also agree with the findings of the present study. 

The present investigation at three locations showed a wide range of variations for different traits. This variation indicated that there is a way to identify promising genotypes based on the traits. The PCV and GCV for all the genotypes in multiple locations were divided into three categories (above 20% was high, 10–20% was medium, and below 10% was low). At the Ishwardi and Jashore locations, high levels of PCV and GCV were found for the ASI. On the other hand, only PCV was high. Moderate levels of PCV and lower levels of GCV were recorded for the EH, KPE, TKW, and GY traits only for the Barishal location. At the Ishwardi location, the PCV of KPE, TKW, and GY were observed at a medium level; however, the EH was at low level for this location. However, the PCV and GCV were moderate for EH and TKW at the Barishal location. In this location, PCV were also moderate for KPE and GY, but the GCV was recorded as low. Both GCV and PCV were at lower levels for AD, SD, and PH in all three locations (Table 1). A medium level of the coefficient of variation implies an equal influence of additive and nonadditive gene action. Medium levels of genotypic coefficients of variance were found for some traits, as reported by several earlier findings [4,31,32]. 

In the present investigation, the PCV was comparatively higher than the GCV for all the traits, but the ranges of difference between the PCV and GCV were low for AD, SD, and PH at the three locations, indicating the low impact of the environment on the expression of the traits, a symptom of the heritable nature of the traits. Several researchers have also observed a similar but higher PCV than GCV in their studies [33,34]. In our study, closer GCV and PCV were found for EH, KPE, TKW, and GY at the Ishwardi and Jashore locations, indicating low environmental influence on the expression of the traits. Therefore, a huge scope for the perfect selection of traits existed based upon the phenotypic expression of these traits. Similar findings were also observed by [35]. The GCV and PCV values were also close in the cases of AD, SD, and PH at all three locations, but the low levels of GCV and PCV (<10%) were not suitable for selection. A wider PCV and GCV value was observed for ASI at the three locations. At the Barishal location, wider GCV and PCV values were also observed for EH, KPE, TKW, and GY, indicating the dominant role of the environment on the expression of the traits, which was not suitable for effective selection. A high level of environmental influence was also found by Patil et al. [35] in the case of some traits. 

### 3.2. Heritability and Genetic Advance

Heritability is a tool that is used to estimate the degree of variation in a group population. The heritability in a group of the population can be classified into three groups (i.e., >80% is high, 40–80% is medium, and low is <40%). In the present investigation, at the Ishwardi location, high heritability was observed for all the traits. For the AD, SD, EH, and TKW traits, heritability was shown to be high at the Jashore location. However, a moderate level of heritability was found for all the traits except the ASI at the Barishal location. At the Jashore location, medium levels of heritability were observed for ASI, PH, KPE, and GY (Table 1). Several earlier findings have revealed that a high level of heritability for any trait indicates a low level of environmental impact on genotypes. The information from the current study related to heritability is helpful for selecting the best traits for the improvement of crops [9,36,37]. The current study also reported a high level of heritability for different traits of maize. However, only heritability-based trait selection may not be successful sometimes, as the broad sense of heritability counts on total genetic variance, which involves additive, dominant, and epistatic variances. Therefore, estimation of the heritability of a group of genotypes coupled with high genetic advance is more reliable and efficient for the selection of desirable traits for a group of the population [38]. According to the categorization of genetic advance as a percentage of mean (GAM) <10 is low, 10–20% is medium, and >20% is high. High heritability coupled with high genetic advances were found in almost all the traits, except ASI and GY at the Ishwardi location. At the Jashore location, high heritability with high genetic advance was also observed for EH and TKW (Table 1). These findings are in accordance with a previous study [39]. High heritability with moderate genetic advance was also observed for AD and SD at the Ishwardi location, which indicates influence from dominance or epitasis. Similar findings have been observed by [4,28]. Moderate heritability with high genetic advance were observed for PH, EH, KPE, and TKW at the Barishal location; on the other hand, at the Jashore location, it was observed for EH and KPE. Moderate heritability coupled with moderate genetic advance was found for AD and SD at the Barishal location. Low estimates of genetic gain were revealed for grain yield (GY) at all the locations, except for the AD and SD value, which showed low genetic gain at the Jashore location. The traits showed high heritability coupled with high genetic advance, which gave information to select superior genotypes. These traits governed by additive gene action would be favorable for a breeding program. 

### 3.3. Association of Traits among Genotypes

Polygenic traits are sensitive to environmental influence. Therefore, the selection of promising genotypes based on only yield may not be effective. For yield improvement or plant architecture improvement, selection has to be performed through associated traits. In our study, correlation coefficient analyses for seven traits were performed at both the phenotypic and genotypic levels (Table 2, Table 3 and Table 4). At three locations, a higher genotypic correlation coefficient was found than the phenotypic correlation coefficient in the present investigation, while a strong inborn association was decreased at the phenotypic level due to environmental effects. The same type of results was also observed [40]. At the Barishal and Jashore locations, the results indicated that some yield-contributing traits, such as PH, EH, and TKW, had a significant positive correlation at the phenotypic, as well as genotypic, level with maize grain yield (Table 2, Table 3 and Table 4). A significant and positive correlation was also observed for PH and EH with grain yield at the Ishwardi location (Table 3). At the Barishal location, the highest value of positive and significant correlation was found between AD and SD (0.99 and 0.92), closely followed by EH and PH (0.99 and 0.70) and GY and PH (0.99 and 0.51). TKW and KPE (−0.77 and −0.31) showed the highest value of significant negative correlation. A significant correlation was found between AD and SD (0.97 and 0.96), closely followed by EH and PH (0.78 and 0.75); on the other hand, ASI and AD (−0.48 and −0.44) exhibited the highest value of negative and significant correlation at the Ishwardi location (Table 3). At the Jashore location, a significant correlation was found between AD and SD (0.99 and 0.95), closely followed by EH and PH (0.84 and 0.81); on the other hand, TKW and KPE (−0.81 and −0.54) exhibited the highest value of negative and significant correlation (Table 4). The recent findings agree with several research observations on different traits of maize [8,35,41]. Hence, selection based on these traits will result in improving the grain yield of maize.

The association of traits as measured by the correlation coefficient may not always show a perfect picture of the relationships among traits. In this way, the regression coefficient helps to examine the relationships of traits and to identify the relative importance of each in contributing to the yield. The regression analysis showed that ASI, PH, KPE, and TKW were effective traits for yield at the Barishal location, and the regression value was 0.42; KPE and TKW were effective traits at the Ishwardi location, and a value of 0.47 contributed towards yield. AD, ASI, EH, KPE, and TKW were effective for the yield of maize at the Jashore location, and the regression value was found to be 0.57 for these traits (Table 5). 

**Figure 1:**
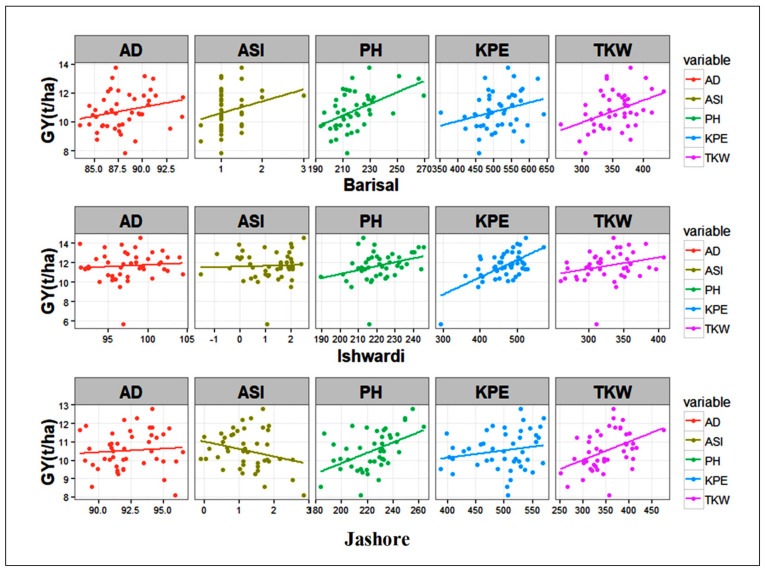
Graph displaying contributions of different traits to the grain yield variation.

Figure 1 displays the contributions of different traits to the grain yield variation. In order to obtain the highest grain yields for different genotypes, the improvement of these traits could obtain a high yield of maize [42]; these traits were used in a stepwise regression in which the grain yield was a dependent variable against other traits as the independent variables [43]. Among the independent variables, TKW, KPE, and PH were the most important traits contributing to the final grain yield of maize. 

The initial model and final models for different locations obtained from the stepwise regression were as follows:

Initial model: GY~AD + SD + ASI + PH + EH + KPE + TKW.

Final model for Barishal: GY~ASI + PH + KPE + TKW.

Final model for Ishwardi: GY~KPE + TKW.

Final model for Jashore: GY~AD + ASI + EH + KPE + TKW.

### 3.4. Genetic Diversity

Cluster analysis is a perfect biometrical tool for grouping data according to similarity. Data can be categorized into homogenous and distinct groups with cluster analysis. In the current study, all the genotypes were classified into four different clusters, with cluster IV having the maximum genotypes (16, 35.55%), followed by cluster II and cluster I (13 and 12, respectively) genotypes (Table 6). 

**Figure 2:**
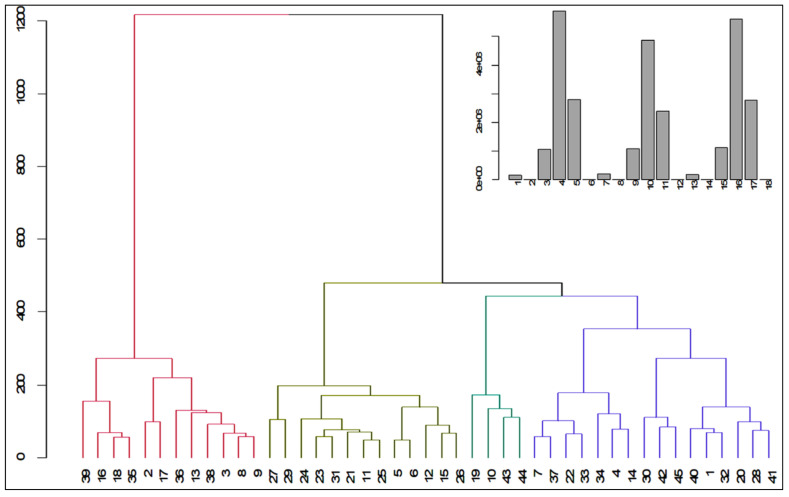
Dendrogram showing the grouping of genotypes based on AD, ASI, PH, KPE, TKW, and GY traits of all locations.

Cluster III contained the lowest number of genotypes (8.88%) and occupied the lowest rank. Remarkably, cluster I had the (G39, G16, G18, G35, G2, G47, G36, G13, G33, G3, G8, and G9) genotypes, whereas cluster II had the (G27, G29, G24, G23, G31, G21, G11, G25, G5, G6, G12, G15, and G26) genotypes. Furthermore, cluster III had (G19, G10, G13, and G44), and cluster IV showed sixteen genotypes: (G7, G37, G22, G33, G34, G4, G14, G45, G40, G1, G32, G20, G28, and G41) (Table 6 and Figure 2). Similar observations have also been found [34,44]. Some parents of the genotypes collected from the same or nearby locations did not fall in the same cluster, informing that geographical closeness did not always give better genetic uniformity. Therefore, there might be underlying factors playing a role behind the genetic differences among genotypes originating from the same areas that may have various genetic make-ups. The dendrogram represents the index of genetic diversity among the clusters and genotypes (Figure 2). Inter- and intracluster distances informed us that there was existing diversity among genotypes. In the case of maize, ref. [45] also found similar findings. By using a covariance matrix in the case of maize nonhierarchical clustering, ref. [46] observed clusters, and ref. [45] also found clusters from maize advance lines.

**Figure 3:**
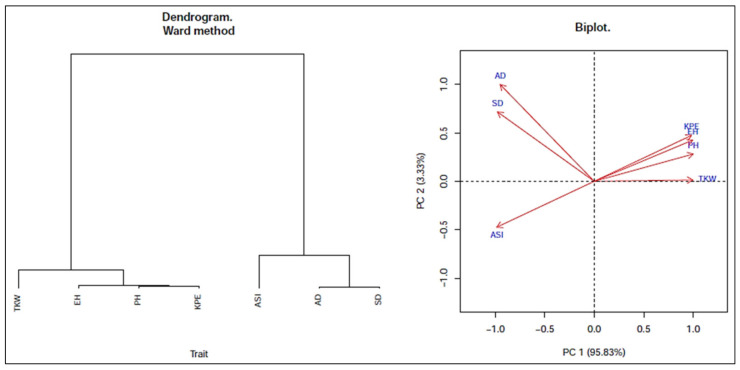
Dendrogram showing clustering of different traits (**left**); position of different traits depicted on biplot from principal component analysis of combined data.

The biplot depicts the positions of different studied traits except for GY and their clustering patterns based on the traits’ weights (Figure 3). The biplot reveals that the studied traits could broadly be classified into two groups. The traits of SD, AD, and ASI were in one set, whereas TKW, KPE, PH, and EH were in the other one. It was found that the traits in such groups looked to be the same type. The PCA explained the partitioning of total variation into principal components (PCs). In the analysis, across the locations, PC1 accounted for 95.83% of the total variation, and PC2 contributed only 3.33%. At the Barishal location, the biplot reveals similar results, as across one plot, the traits were majorly grouped into two, and in the other, they were sub-grouped into four: traits KPE, PH, and EH were together in a subgroup with AD and SD in one subgroup, ASI in another subgroup, and lastly, TKW was the furthest in the fourth subgroup. At this location, PC1 accounted for 94.83% and PC2 for 4.56% of the total variability (Figure S1). At the Ishwardi location, PC1 contributed 80.98%, while PC2 accounted for 16.3% of the total diversity (Figure S2). The biplot depicts that the traits of PH and EH, as well as KPE and TKW, were included largely in one group together, whereas AD, SD, and ASI were in another group, following a similar pattern as before. At the Jashore location, the biplot shows the traits of PH, EH, KPE, and TKW together into a major group, whereas AD, SD, and ASI were in another one. The PC1 accounted for 72.71% of the variability, and PC2 accounted for 23.9% of the total diversity (Supplementary Figure S3). PCA analysis accounted for the traits and variables and reduced them into PCs, where in the present study, the most variability (~99%) was conferred by the first two PCs. Similar findings have been observed in cases of maize by [11,47].

### 3.5. Genotype × Location Interaction Analysis

A genotype × location interaction analysis was performed based on grain yield observations at different locations. Ample variation (*p* ≤ 0.01) was observed for the studied genotypes in the combined analysis of variance (Table 7), which indicated the differential responses of genotypes at different locations. 

The highest portion (46.004%) of the total sum of squares was explained by the genotypic effect, which indicated the presence of ample genetic variability among the studied genotypes and the possibility of selection for stable, high-yielding genotypes. The location was the least source of variation and contributed only a small portion (11.286%) to the total sum of squares. Moreover, location was not significant, indicating that across the genotypes, the means at locations were not varied statistically. However, a significant difference was spotted for genotype × location interaction, suggesting that the grain yield of genotypes varied across the locations and reflecting the existence of locational effects in the genotype × location interaction. A high percentage (42.709%) of the total sum of squares for the genotype × location interaction displayed the significance of this source of variation and also implicated a truncated effectiveness of indirect selection for potential genotypes disregarding the genotype × location interaction. Genotype–location interactions had a role in the stability of the tested genotypes. Therefore, the stability of the genotypes was measured because the difference in locations accounted for most of the population [48,49,50]. Parag et al. [51] also found significant variation due to a genotype × location interaction for the yield of maize. Hence, a significant genotype × location interaction may influence crop development, which a plant breeder can use in a maize variety development program if genotypes are to be adopted to explicit climates.

### 3.6. Top Yielder at Locations

The yield of maize under the investigation varied with locations. The mean grain yield of the genotypes over the locations ranged from 8.46 t/ha to 12.70 t/ha with a total mean of 11.02 t/ha (Table 8). Quite a few genotypes showed wider adaptation, but some also showed region-centric better performances. Overall, genotypes G5, G8, G27, G29, and G42 were the top five based on yield performance for wider adaption, in which genotype G42 ranked first. At the Barishal location, the highest value for yield was 13.79 t/ha, and the lowest value was 7.80 t/ha. The best-performing five genotypes at this location were G8, G29, G30, G42, and G45. G42 was the top performer here, whereas G8 and G45 showed somewhat region-specific yielders compared to the other two locations. The maximum value for yield was found to be 14.51 t/ha, and the minimum value was 5.68 t/ha at Ishwardi. The best five performers were the G5, G7, G13, G27, and G37 genotypes, of which G37 was the top yielder. At this particular location, most of the top-yielding genotypes were region-centric in nature, except G5 and G27. On the other hand, the maximum value for yield was 12.89 t/ha, and the minimum value was 8.27 t/ha at the Jashore location. The five best-yielding genotypes were G28, G29, G30, G41, and G42, and G29 was the highest yielder. The region-specific adaption was observed for genotype G28. The experimental locations were ranked based on yield potential as Ishwardi > Barishal > Jashore. The present findings showed differential yield potentials at the three locations. The locations of Barishal, Jashore, and Ishwardi were also distinct and suggested the existence of genotype–location interactions. Similar observations have been found [52,53]. A vast majority of the genotypes showed unstable performances among the locations. In a study with maize genotypes, Badu-Apraku et al. [54] found some high-yielding, unstable genotypes in West Africa. In the present study, a few other genotypes exhibited near-perfect performances, i.e., better yielding ability across the locations. A perfect genotype must have a high mean value of yield and a high level of stability in vast environments [53,55].

## 4. Conclusions

Improvement of maize yield-contributing traits was possible using phenotypic selection for PH, ASI, EH, and TKW, of which all locations showed high values for the genotypic and phenotypic coefficients of variation coupled with h^2^b (heritability) and GA (genetic advance). These traits also revealed positive or negative direct effects on maize yield. Therefore, priority should be given to these traits for crop improvement. The diversity analysis provided a way to choose the best recombinants for different traits and further create variations in these traits in future segregants. Hence, traits controlled by additive gene action may be amenable to a breeding program. These traits can be used for the improvement of maize through selection. Quite a few genotypes were found to be better either for wider adaption or specific to a particular location, which needs to be further validated before recommending for cultivation.

## Figures and Tables

**Table 1:** Estimation of genetic parameters in eight traits of 45 genotypes of maize grown in multiple locations.

Traits	X	LSD	CV%	*σ* ^2^ *_g_*	*σ* ^2^ *_p_*	*σ* ^2^ *_e_*	GCV	PCV	ECV	h^2^	GA
Barishal location											
AD	88.03	4.40	2.37	4.10 **	8.44	4.34	2.30	3.30	24.69	0.65	11.37
SD	89.12	4.78	2.60	3.47 *	8.85	5.38	2.09	3.34	26.22	0.56	10.26
ASI	1.20	1.34	52.07	0.00 ^ns^	0.39	0.39	0.00	52.07	160.05	0.00	0.00
PH	218.06	36.99	8.08	111.73 ^ns^	422.02	310.29	4.85	9.42	4.17	0.42	363.96
EH	119.18	33.00	13.56	129.77 *	390.86	261.09	9.56	16.59	4.13	0.50	401.39
KPE	510.99	123.35	11.18	1288.16 ^ns^	4551.01	3262.85	7.02	13.20	1.26	0.44	4136.42
TKW	352.83	74.45	10.33	578.54 *	1907.60	1329.07	6.82	12.38	1.91	0.47	1828.90
GY	10.75	2.61	11.87	0.90 *	2.53	1.63	8.83	14.79	50.46	0.53	2.74
Ishwardi location											
AD	97.40	1.64	0.82	11.25 **	11.89	0.64	3.44	3.54	6.75	0.97	23.82
SD	98.51	1.69	0.83	9.47 **	10.13	0.67	3.12	3.23	8.05	0.97	20.16
ASI	1.12	0.99	39.18	0.67 **	0.86	0.19	73.22	83.04	50.77	0.87	1.56
PH	220.95	16.42	3.34	103.48 **	157.90	54.42	4.60	5.69	4.67	0.79	257.54
EH	115.04	15.02	5.90	78.14 **	124.15	46.01	7.68	9.69	5.46	0.77	197.59
KPE	465.02	50.52	5.12	2258.45 **	2826.25	567.79	10.22	11.43	0.84	0.89	5171.93
TKW	326.60	23.13	3.47	1170.97 **	1299.27	128.30	10.48	11.04	0.87	0.95	2537.49
GY	11.65	1.64	6.79	1.84 **	2.47	0.63	11.65	13.48	32.04	0.85	4.35
Jashore location											
AD	92.33	1.38	0.72	4.13 **	4.57	0.44	2.20	2.32	14.58	0.95	8.94
SD	93.55	1.90	0.92	4.41 **	5.16	0.75	2.24	2.43	16.78	0.92	9.79
ASI	1.22	1.48	53.54	0.18 ^ns^	0.61	0.43	34.37	63.62	108.06	0.45	0.56
PH	224.18	28.95	5.85	222.41 **	394.22	171.81	6.65	8.86	3.32	0.72	585.82
EH	115.23	12.45	4.57	215.42 **	243.16	27.74	12.74	13.53	2.17	0.94	470.62
KPE	497.40	81.36	8.01	1776.91 **	3363.96	1587.05	8.47	11.66	1.18	0.69	4790.46
TKW	351.33	55.03	7.62	1758.71 **	2476.29	717.57	11.94	14.16	1.08	0.83	4236.82
GY	10.51	2.05	9.16	0.60 *	1.53	0.93	7.38	11.76	62.97	0.57	1.78

* 5% level of probability, ** 1% level of probability; ^ns^ non-significant; AD: days to anthesis, SD: days to silking, ASI: anthesis–silking interval, PH: plant height, EH: ear height, KPE: kernels per plant, TKW: thousand-kernel weight, GY: grain yield, X: mean value, LSD: least significant difference, CV%: coefficient of variation, *σ*^2^*_g_*: genotypic variance, *σ*^2^*_p_*: phenotypic variance, *σ*^2^*_e_*: environmental variance, GCV: genotypic coefficient of variation, PCV: phenotypic coefficient of variation, ECV: environmental coefficient of variation, h^2^: heritability, GA: genetic advance.

**Table 2:** Associations of different traits from trial evaluated at Barishal location.

Traits		AD	SD	ASI	PH	EH	KPE	TKW
SD	r_g_	0.99 **						
	r_p_	0.92 **						
ASI	r_g_	-	-					
	r_p_	0.06	0.02					
PH	r_g_	0.80 **	0.74 **	-				
	r_p_	0.43 **	0.39 *	0.07				
EH	r_g_	0.36 *	0.27	-	0.99 **			
	r_p_	0.32 *	0.30 *	0.17	0.70 **			
KPE	r_g_	0.17	0.22	-	0.62 **	0.35 *		
	r_p_	0.23	0.27	0.21	0.29	0.18		
TKW	r_g_	−0.33 *	−0.19	-	0.23	24	−0.77 **	
	r_p_	−0.19	−0.11	−0.04	0.13	0.15	−0.31 *	
GY	r_g_	0.43 **	0.40 *	-	0.99 **	0.73 **	0.61 **	0.48 **
	r_p_	0.24	0.23	0.26	0.51 **	0.42 **	0.28	0.41 *

* 5% level of probability, ** 1% level of probability, AD: days to anthesis, SD: days to silking, ASI: anthesis–silking interval, PH: plant height, EH: ear height, KPE: kernels per plant, TKW: thousand kernel-weight, GY: grain yield, r_g_: genotypic correlation coefficient, r_p_: phenotypic correlation coefficient.

**Table 3:** Associations of different traits from trial evaluated at Ishwardi location.

Traits		AD	SD	ASI	PH	EH	KPE	TKW
SD	r_g_	0.97 **						
	r_p_	0.96 **						
ASI	r_g_	−0.48 **	−0.29					
	r_p_	−0.44 **	−0.18					
PH	r_g_	0.2	0.21	−0.06				
	r_p_	0.15	0.18	0.04				
EH	r_g_	0.43 **	0.53 **	0.2	0.78 **			
	r_p_	0.25	0.35 *	0.24	0.75 **			
KPE	r_g_	0.32 *	0.37 *	0.13	0.23	0.46 **		
	r_p_	0.28	0.32 *	0.04	0.26	0.34 *		
TKW	r_g_	−0.16	−0.16	0.04	0.04	0.2	−0.19	
	r_p_	−0.15	−0.16	0.03	0.08	0.14	−0.17	
GY	r_g_	0.09	0.13	0.02	0.36 *	0.52 **	0.50 **	0.27
	r_p_	0.08	0.11	0.05	0.33 *	0.37 *	0.62	0.28

* 5% level of probability, ** 1% level of probability, AD: days to anthesis, SD: days to silking, ASI: anthesis–silking interval, PH: plant height, EH: ear height, KPE: kernels per plant, TKW: thousand-kernel weight, GY: grain yield, r_g_: genotypic correlation coefficient, r_p_: phenotypic correlation coefficient.

**Table 4:** Associations of different traits from the trial were evaluated at the Jashore location.

Traits		AD	SD	ASI	PH	EH	KPE	TKW
SD	r_g_	0.99 **						
	r_p_	0.95 **						
ASI	r_g_	0.04	0.24					
	r_p_	−0.03	0.27					
PH	r_g_	0.54 **	0.57 **	0.30 *				
	r_p_	0.42 **	0.41 *	0.00				
EH	r_g_	0.61 **	0.59 **	0.15	0.84 **			
	r_p_	0.53 **	0.54 **	0.07	0.81 **			
KPE	r_g_	0.69 **	0.71 **	0.27	0.63 **	0.58 **		
	r_p_	0.56 **	0.55 **	0.05	0.42 **	0.43 **		
TKW	r_g_	−0.32 *	−0.29	0.15	0.26	0.10	−0.81 **	
	r_p_	−0.27	−0.25	0.04	0.12	−0.01	−0.54 **	
GY	r_g_	0.10	0.03	−0.38 *	0.85 **	0.78 **	−0.05	0.54 **
	r_p_	0.08	0.00	−0.26	0.49 **	0.42 **	0.20	0.44 **

* 5% level of probability, ** 1% level of probability, AD: days to anthesis, SD: days to silking, ASI: anthesis–silking interval, PH: plant height, EH: ear height, KPE: kernels per plant, TKW: thousand-kernel weight, GY: grain yield, r_g_: genotypic correlation coefficient, r_p_: phenotypic correlation coefficient.

**Table 5:** Initial model and final models for different locations were obtained from stepwise regression.

Traits		AD	SD	ASI	PH	EH	KPE	TKW	Multiple Regression	Stepwise Regression
Barishal	b	0.122	0.121	0.820	0.040	0.030	0.006	0.015		
	r^2^	0.056	0.053	0.070	0.256	0.170	0.070	0.167	0.42	0.45
	*p* *-value*	0.116	0.120	0.082	0.000	0.004	0.060	0.005	<0.000	<0.000
Ishwardi	b	0.036	0.050	0.070	0.040	0.046	0.017	0.012		
	r^2^	0.006	0.011	0.001	0.106	0.133	0.380	0.070	0.47	0.51
	*p* *-value*	0.587	0.486	0.774	0.029	0.010	<0.000	0.060	<0.000	<0.000
Jashore	b	0.038	0.000	−0.400	0.028	0.027	0.004	0.009		
	r^2^	0.006	0.000	0.060	0.236	0.176	0.038	0.193	0.57	0.59
	*p* *-value*	0.608	0.990	0.080	0.001	0.004	0.190	0.002	<0.000	<0.000

AD: days to anthesis, SD: days to silking, ASI: anthesis–silking interval, PH: plant height, EH: ear height, KPE: kernels per plant, TKW: thousand-kernel weight, b: slope, r^2^: coefficient of determination.

**Table 6:** Cluster analysis of forty-five maize genotypes.

Cluster	Number of Genotypes	Percentage (%)	Accession Number
I	12	26.66	G39, G16, G18, G35, G2, G47, G36, G13, G33, G3, G8, G9
II	13	28.88	G27, G29, G24, G23, G31, G21, G11,G25, G5, G6, G12, G15, G26
III	4	8.88	G19, G10, G13,G44
IV	16	35.55	G7,G37, G22, G33, G34, G4, G14, G30, G42, G45, G40, G1, G32, G20, G28, G41

**Table 7:** Genotype–location interaction ANOVA for grain yield studied at three locations.

Source of Variation	Degrees of Freedom	Sum Squares	Mean Squares	% Total SS
Location	2	53.901	26.951	11.286
Genotype	44	219.712	4.993 **	46.004
Genotype × Location	88	203.979	2.318 **	42.709
Residuals	132	139.081	1.0536	-

** 1% level of probability.

**Table 8:** Mean grain yield performances of the studied genotypes at different locations.

Gen	Bar	Ish	Jas	Mean	Pi	Gen	Bar	Ish	Jas	Mean	Pi
G1	11.12	11.35	11.43	11.30	0.28	G24	10.54	10.17	10.61	10.44	−0.58
G2	10.82	12.53	11.00	11.45	0.43	G25	10.20	11.88	9.57	10.55	−0.48
G3	9.78	12.05	10.10	10.64	−0.38	G26	11.86	13.19	9.94	11.66	0.64
G4	10.38	11.54	8.53	10.15	−0.87	G27	12.18	13.62 *	10.44	12.08 *	1.06
G5	11.68	13.58 *	11.53	12.26 *	1.24	G28	10.58	11.92	12.07 *	11.52	0.50
G6	11.46	11.51	9.61	10.86	−0.16	G29	13.00 *	11.84	12.89 *	12.57 *	1.55
G7	11.51	13.91 *	9.90	11.77	0.75	G30	13.19 *	10.37	12.15 *	11.90	0.88
G8	13.04 *	12.51	11.39	12.31 *	1.29	G31	8.63	13.09	9.14	10.28	−0.74
G9	12.13	12.91	10.88	11.97	0.95	G32	11.57	11.31	10.73	11.20	0.18
G10	9.81	10.56	8.27	9.55	−1.48	G33	10.59	13.05	11.24	11.62	0.60
G11	9.79	11.53	10.32	10.55	−0.48	G34	9.22	11.32	10.18	10.24	−0.78
G12	9.35	10.10	9.92	9.79	−1.23	G35	9.73	12.65	11.04	11.14	0.12
G13	10.83	13.86 *	10.12	11.60	0.58	G36	10.48	11.99	11.40	11.29	0.27
G14	9.80	11.38	10.88	10.68	−0.34	G37	9.70	14.51 *	10.65	11.62	0.60
G15	11.21	11.62	11.39	11.41	0.39	G38	8.78	12.52	10.97	10.75	−0.27
G16	9.51	10.02	9.60	9.71	−1.31	G39	10.62	11.14	10.75	10.83	−0.19
G17	10.62	10.63	11.22	10.82	−0.20	G40	9.53	11.39	11.19	10.70	−0.32
G18	10.33	10.68	9.76	10.26	−0.76	G41	11.80	11.99	12.11 *	11.97	0.95
G19	9.75	5.68	9.97	8.46	−2.56	G42	13.79 *	12.35	11.97 *	12.70 *	1.68
G20	10.56	10.83	10.92	10.77	−0.25	G43	7.80	9.48	9.67	8.98	−2.04
G21	11.83	10.57	11.05	11.15	0.13	G44	9.12	10.83	10.13	10.03	−0.99
G22	12.24	12.38	10.26	11.63	0.61	G45	12.31 *	10.32	11.79	11.47	0.45
G23	11.17	11.71	11.10	11.32	0.30						
Mean	10.75	11.65	10.66	11.02		Mean	10.75	11.65	10.66	11.02	
Li	−0.27	0.63	−0.36			Li	−0.27	0.63	−0.36		

Gen: genotype; Bar: Barishal; Ish: Ishwardi; Jas: Jashore; Pi: phenotypic index; Li: locational index; * indicates the five best-performing genotypes in terms of grain yield; Significant at 5% level of probability.

## Data Availability

Not applicable.

## Supplementary Materials

The following supporting information can be downloaded at: https://www.mdpi.com/2223-7747/11/11/1522/s1”. Table S1: Details of the studied genotypes; Table S2: Weather conditions prevailing for vegetative and reproductive phases during cropping period at different locations; Figure S1: Dendrogram showing clustering of different traits (left); the position of different traits depicted on biplot from principal component analysis for data on Barishal environment; Figure S2: Dendrogram showing clustering of different traits (left); the position of different traits depicted on biplot from principal component analysis for data on Ishwardi environment; Figure S3: Dendrogram showing clustering of different traits (left); position of different traits depicted on biplot from principal component analysis for data on Jashore environment.
