# Supplementary material for: Genetic Variation and Genotype by Environment Interaction for Agronomic Traits in Maize (Zea mays L.) Hybrids
Source: Plants (Basel). 2022 Jun 6;11(11):1522. doi: 10.3390/plants11111522 (PMC9182618; doi:10.3390/plants11111522)
Supplement: Supplementary file 1 [file plants-11-01522-s001.zip › plants-1638252-supplementary.pdf]

Table S1: Details of the studied genotypes

Hybrid code	Pedigree
G1	BD-ISD-IL-E-8-B//BIL-22-B3
G2	BD-ISD-IL-E-8-B//BIL-28-B3
G3	BD-ISD-IL-E-8-B//BIL-79-B3
G4	BD-ISD-IL-E-16-B//BIL-22-B3
G5	BD-ISD-IL-E-16-B//BIL-28-B3
G6	BD-ISD-IL-E-16-B//BIL-79-B3
G7	BD-ISD-IL-E-18-B//BIL-22-B3
G8	BD-ISD-IL-E-18-B//BIL-28-B3
G9	BD-ISD-IL-E-18-B//BIL-79-B3
G10	BD-ISD-IL-E-19-B//BIL-22-B3
G11	BD-ISD-IL-E-19-B//BIL-28-B3
G12	BD-ISD-IL-E-19-B//BIL-79-B3
G13	BD-ISD-IL-E-22-B//BIL-22-B3
G14	BD-ISD-IL-E-22-B//BIL-28-B3
G15	BD-ISD-IL-E-22-B//BIL-79-B3
G16	BD-ISD-IL-E-24-B//BIL-22-B3
G17	BD-ISD-IL-E-24-B//BIL-28-B3
G18	BD-ISD-IL-E-24-B//BIL-79-B3
G19	BD-ISD-IL-E-25-B//BIL-22-B3
G20	BD-ISD-IL-E-25-B//BIL-28-B3
G21	BD-ISD-IL-E-25-B//BIL-79-B3
G22	BD-ISD-IL-E-29-B//BIL-22-B3
G23	BD-ISD-IL-E-29-B//BIL-28-B3
G24	BD-ISD-IL-E-29-B//BIL-79-B3
G25	BD-ISD-IL-E-34-B//BIL-22-B3
G26	BD-ISD-IL-E-34-B//BIL-28-B3
G27	BD-ISD-IL-E-34-B//BIL-79-B3
G28	BD-ISD-IL-E-43-B//BIL-22-B3
G29	BD-ISD-IL-E-43-B//BIL-28-B3
G30	BD-ISD-IL-E-43-B//BIL-79-B3
G31	BD-ISD-IL-E-44-B//BIL-22-B3
G32	BD-ISD-IL-E-44-B//BIL-28-B3
G33	BD-ISD-IL-E-44-B//BIL-79-B3
G34	BD-ISD-IL-E-45-B//BIL-22-B3
G35	BD-ISD-IL-E-45-B//BIL-28-B3
G36	BD-ISD-IL-E-45-B//BIL-79-B3
G37	BD-ISD-IL-E-46-B//BIL-22-B3
G38	BD-ISD-IL-E-46-B//BIL-28-B3
G39	BD-ISD-IL-E-46-B//BIL-79-B3
G40	BD-ISD-IL-E-54-B//BIL-22-B3
G41	BD-ISD-IL-E-54-B//BIL-28-B3
G42	BD-ISD-IL-E-54-B//BIL-79-B3
G43	BD-ISD-IL-E-57-B//BIL-22-B3
G44	BD-ISD-IL-E-57-B//BIL-28-B3
G45	BD-ISD-IL-E-57-B//BIL-79-B3

Table S2: Weather condition prevailed at vegetative and reproductive phase during cropping period at different locations

Site	Crop phase	Item	Temperature (°C)		Relative Humidity (%)		Rainfall (mm)	Rainy day
			Min	Max	Min	Max		
Ishwardi	Veg.	Mean	19.37	33.8	33.8	96.6	0	0
		Max	23.6	35.6	50	100		
		Min	16.3	31	24	89		
	Rep.	Mean	20.86	35.65	27.87	90.93	1.3	3
		Max	22.8	37.5	50	100		
		Min	17.4	32.8	18	75		
Jashore	Veg.	Mean	16.19	33.07	48.07	65.53	0	0
		Max	22	35	64	90		
		Min	12.6	30.4	40	47		
	Rep.	Mean	21.53	35.32	52.2	75.8	8	1
		Max	23.8	37.8	69	93		
		Min	16.2	33.2	32	51		
Borishal	Veg.	Mean	34.04	15.95	36.2	93.4	0	0
		Max	35.6	18	42	97		
		Min	32	14.4	31	87		
	Rep.	Mean	35.97	21.52	47.47	93.53	1.4	1
		Max	38.2	24	57	95		
		Min	32	17.4	41	90		

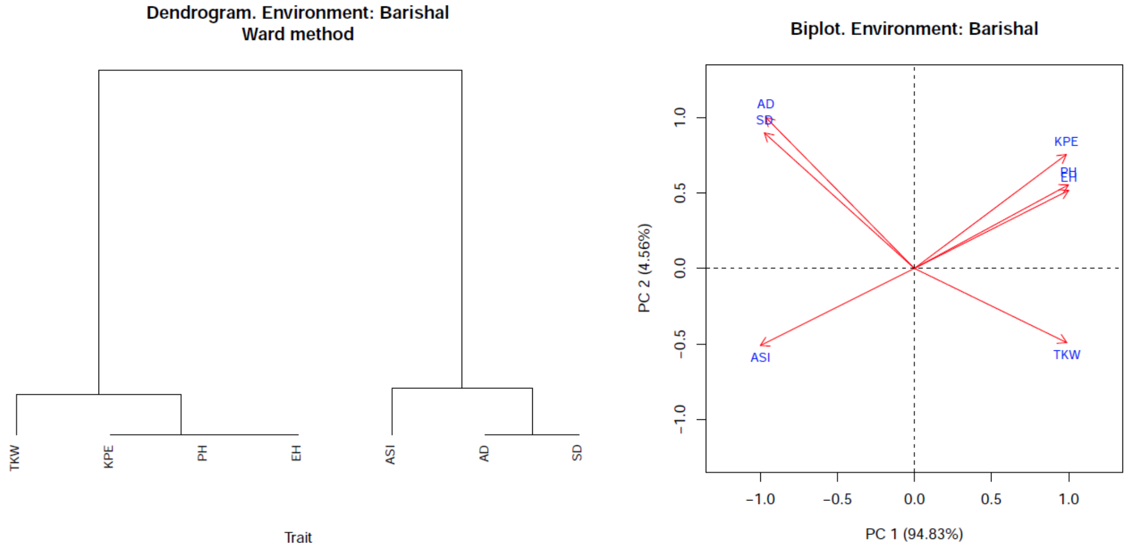

Figure S1. Dendrogram showing clustering of different traits (left); position of different traits depicted on biplot from principle component analysis on data of Barishal environment

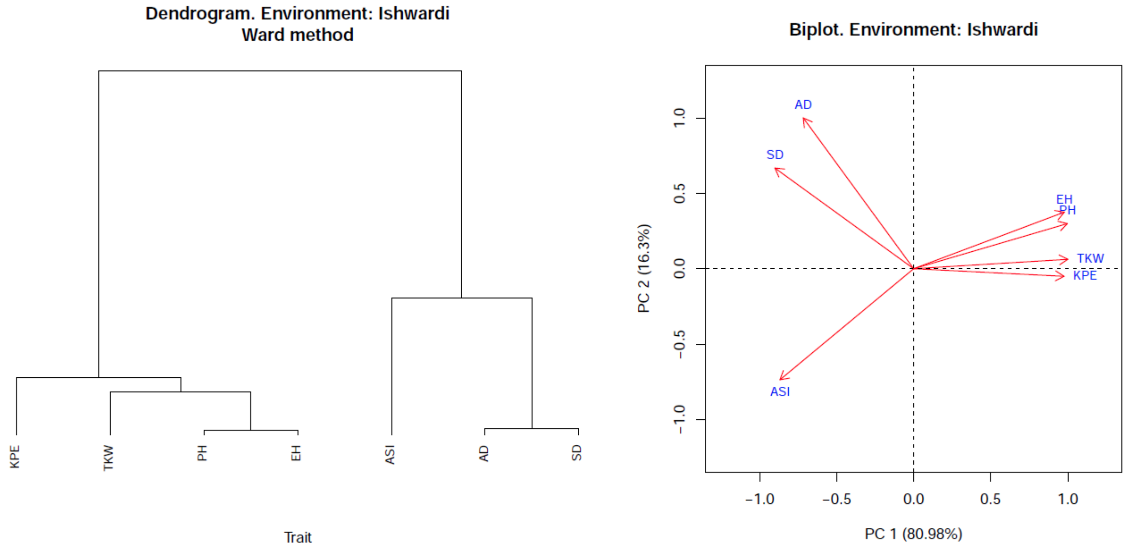

Figure S2. Dendrogram showing clustering of different traits (left); position of different traits depicted on biplot from principle component analysis on data of Ishwardi environment

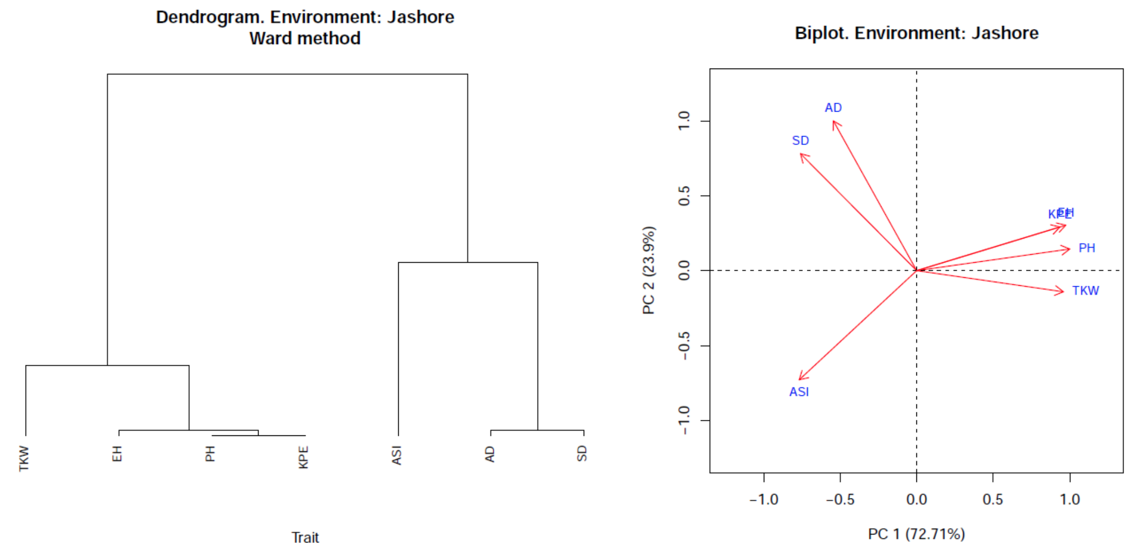

Figure S3. Dendrogram showing clustering of different traits (left); position of different traits depicted on biplot from principle component analysis on data of Jashore environment
